# O.2.1-10 Intergenerational leisure-time physical activity across three generations in Finland

**DOI:** 10.1093/eurpub/ckad133.115

**Published:** 2023-09-11

**Authors:** Xiaolin Yang, Tuomas Kukko, Suvi Rovio, Katja Pahkala, Janne Kulmala, Olli Raitakari, Tuija Tammelin

**Affiliations:** Likes, School of Health and Social Studies, Jamk University of Applied Sciences, Jyväskylä, Finland; Likes, School of Health and Social Studies, Jamk University of Applied Sciences, Jyväskylä, Finland; Research Centre of Applied and Preventive Cardiovascular Medicine, University of Turku, Turku, Finland; Centre for Population Health Research, University of Turku and Turku University Hospital, Turku, Finland; Research Centre of Applied and Preventive Cardiovascular Medicine, University of Turku, Turku, Finland; Centre for Population Health Research, University of Turku and Turku University Hospital, Turku, Finland; Paavo Nurmi Centre and Unit for Health and Physical Activity, University of Turku, Turku, Finland; Likes, School of Health and Social Studies, Jamk University of Applied Sciences, Jyväskylä, Finland; Research Centre of Applied and Preventive Cardiovascular Medicine, University of Turku, Turku, Finland; Centre for Population Health Research, University of Turku and Turku University Hospital, Turku, Finland; Department of Clinical Physiology and Nuclear Medicine, Turku University Hospital, Turku, Finland; xiaolin.yang@jamk.fi; Likes, School of Health and Social Studies, Jamk University of Applied Sciences, Jyväskylä, Finland

## Abstract

**Purpose:**

Associations of leisure-time physical activity (LTPA) in three-generational families has not been well studied. This study aimed to examine these associations in a three-generational data including children, and their parents and grandparents.

**Methods:**

Self-reported LTPA data, along with a range of family sociodemographic and health-related variables, were extracted from the ongoing Young Finns Study. The cohort consists of 2501 children aged 7–38 years, their parents, and grandparents in 2018. Structural equation modeling was fitted to estimate the associations of children’s LTPA with parents’ and grandparents’ LTPA in different age and sex groups.

**Results:**

Interrelationships of parental and grandparental LTPA with children’s LTPA varied by age and sex groups. For children aged 7–12 years, mothers’ LTPA was associated with higher LTPA in both boys (r = 0.22, p = 0.017) and girls (r = 0.34, p < 0.001), while fathers’ LTPA was associated with higher LTPA in boys (r = 0.25, p = 0.01). High LTPA level in maternal grandfathers and grandmothers was associated with higher LTPA in grandgirls (r = 0.29, p = 0.024 and r = 0.23, p = 0.039). For adolescents aged 13–18 years, only fathers’ LTPA was associated with higher LTPA in boys (r = 0.20, p = 0.029). For young adults aged >18 years, fathers’ LTPA was associated with higher LTPA in both young males (r = 0.21, p = 0.004) and females (r = 0.20, p = 0.003), while mothers’ LTPA was associated with higher LTPA in young females (r = 0.13, p = 0.009). High LTPA level in maternal grandfathers was associated with higher LTPA in grandboys (r = 0.34, p = 0.003).

**Conclusions:**

The children’s LTPA levels associated independently with parents’ and grandparents’ LTPA depending on the child’s age and sex. Given the primary results available, further research is warranted to fully understand the interrelationships between parents' and grandparents’ LTPA and their children’s LTPA in order to create effective and targeted interventions.

